# Gamma knife thalamotomy in treating refractory tremor: initial clinical experience in Croatia

**DOI:** 10.3325/cmj.2024.65.59

**Published:** 2024-02

**Authors:** Slaven Lasić, Sergej Marasanov, Marjan Rožanković, Petra Bago Rožanković

**Affiliations:** 1 Department of Neurology, University Hospital Dubrava, Zagreb, Croatia; 2 Division of Stereotactic, Functional Neurosurgery and Radiosurgery, Department of Neurosurgery, Zagreb University Hospital Center, Zagreb, Croatia; 3 Department of Neurosurgery, Zagreb University Hospital Center, Zagreb, Croatia; 4 School of Medicine, Catholic University of Croatia, Zagreb, Croatia

Tremor refractory to pharmacological therapy significantly reduces the patient´s quality of life, often leading to early retirement and social isolation. Gamma knife (GK) stereotactic radiosurgery of the unilateral thalamic ventral intermediate nucleus is an advanced, minimally invasive surgical procedure for symptomatic tremor suppression. Due to the restricted availability of this type of treatment, literature data on its efficacy and safety are lacking. We present two patients with severe, disabling tremor (one with parkinsonian and one with essential tremor) successfully treated with GK thalamotomy, performed in Croatia for the first time. GK thalamotomy should be considered in patients with refractory tremors and contraindications for deep brain stimulation.

Tremor is an involuntary, rhythmic, oscillatory movement of a body part. Essential tremor is an isolated postural or action tremor of the upper limbs bilaterally, with or without involvement of other locations, that lasts at least three years. The most common acquired tremor syndrome with additional signs is parkinsonian tremor typically manifested as a 4 to 7 Hz rest tremor of the hand, lower limb, foot, jaw, or tongue, sometimes coexisting with postural and action tremor (1).

Patients with severe, medication-refractory tremor undergo invasive treatments including deep brain stimulation (DBS) of the thalamic or subthalamic region, magnetic resonance imaging-guided focused ultrasound lesioning (MRgFUS), radiofrequency thermocoagulation, and gamma knife (GK) radiosurgery (2,3).

GK stereotactic radiosurgery of the unilateral thalamic ventral intermediate (VIM) nucleus is a minimally invasive surgical procedure for symptomatic tremor suppression, especially in patients who have contraindication for DBS (4,5). However, due to the small number of studies, all available data on its efficacy and safety represent valuable contributions to the field.

We present two patients with severe, disabling tremor (one with parkinsonian and one with essential tremor) successfully treated with GK thalamotomy, performed in Croatia for the first time.

## Case reports

The first patient was a 79-year-old, right-handed man with a history of Parkinson’s disease (PD) for the previous eight years (Table 1). The patient was admitted to our infirmary for movement disorders in January 2019. He reported worsening motor symptoms during the previous year, with a prominent disabling tremor in all limbs. Neurological examination in the “off” state revealed right-sided predominant motor symptoms, including severe resting tremor of the head, severe resting, postural, and action tremor of all limbs, with signs of severe bradykinesia and rigor. In the “on” state, all motor symptoms improved, except tremor. The score on the Unified Parkinson´s Disease Rating Scale III in the “off” state was 57 (of which 22 for tremor questions) and 12 in the “on” state (all for tremor questions). The Fahn-Tolosa-Marin tremor rating scale (FTM) score was 86, and the Bain and Findley Tremor Activities of Daily Living (BFT-ADL) scale score was 78. The patient did not have any significant comorbidities. Neuropsychological testing showed mild cognitive impairment and signs of paranoid ideations. Brain magnetic resonance imaging (MRI) was unremarkable.

**Table 1:** The timeline of events and interventions for case 1*

Date	Clinical information
8 years before baseline	The first symptoms of Parkinson’s disease appeared – resting tremor of the right hand, right-sided bradykinesia, and rigor.
1 year before baseline	Worsening of bilateral motor symptoms, appearance of disabling tremor in all limbs even in the “on” phase.
Baseline visit – January 2019	“Off”-phase UPDRS 57 (of which tremor score 22, worse on the right); “on” phase: persisting severe tremor. “On”-phase UPDRS 12 (all tremor scores), FTM 86, BFT-ADL 78. Cognitive and psychiatric symptoms. DBS contraindicated.
6 months later	GK radiosurgery of the left thalamic VIM nucleus – 130 Gy 4 mm isocenter. No complications.
10 months later (4 months after GK)	Follow-up: initial reduction of right sided tremor, no side effects.
14 months later (8 months after GK)	Follow-up: Brain MRI (Figure 1) - adequate VIM lesion, significant reduction of tremor, FTM 19, BFT-ADL 25. No side effects.
37 months later (31 months after GK)	Follow-up: sustained reduction of tremor, FTM 10. No side effects.

*Abbreviations: UPDRS - Unified Parkinson´s Disease Rating Scale; FTM - Fahn-Tolosa-Marin tremor rating scale; BFT-ADL - Bain and Findley tremor activities of daily life scale score; DBS – deep brain stimulation; GK – gamma knife; VIM – ventral intermediate nucleus of the thalamus; MRI – magnetic resonance imaging.

Due to advanced age and cognitive and psychiatric symptoms, DBS was contraindicated. The patient´s major problem was medication-refractory tremor with right-sided dominance. Other motor symptoms were satisfactorily controlled with antiparkinsonian drugs. To treat the right-sided refractory tremor, we decided to perform minimally invasive GK radiosurgery targeting the left thalamic VIM nucleus using 130 Gy with a single isocenter 4 mm shot according to standardized methodology. A single central dose of 130 Gy was applied using a 4-mm collimator with a Leksell Gamma Knife unit, the Icon model (Elekta AB, Stockholm, Sweden). The target localization of VIM was assessed with 1,5 T MRI with 1 mm-thick slices aligned to the anterior commissure-posterior commissure (AC-PC) line. The anterior-posterior coordinate was 9 mm anterior to the PC or 1/3 of the line, 2 mm superior to the AC-PC plane, and 14 mm lateral from the midline reference. A separate targeting was done according to the diagram of Guiot. The shot was finally positioned on the basis of both targeting methods, taking into account the position of the shot in relation to the internal capsule and the width of the third ventricle. The coordinates were co-registered with the atlas of Schaltenbrand and Wahren. The procedure was carried out without complications.

**Figure 1:**
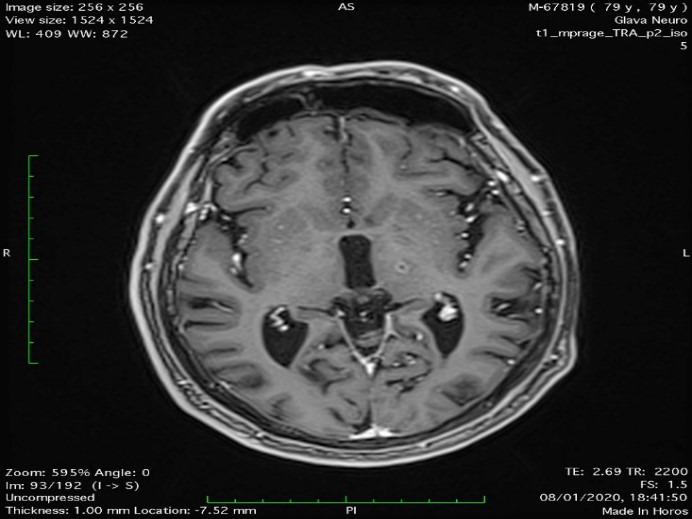
A brain magnetic resonance image (eight months after gamma knife thalamotomy) of case 1 shows an axial plane of a T1, magnetization-prepared rapid gradient echo sequence with adequate irradiation lesion of the left thalamic ventral intermediate nucleus.

On a follow-up four months later, an initial clinical improvement was observed with no side effects reported. The brain MRI performed eight months after the procedure showed an adequate radiation-induced lesion in the left VIM (Figure 1). On neurological examination, tremor was considerably reduced, the FTM score was 19, and the BFT-ADL score improved to 25 points. The patient reported no side effects. On the last follow-up, 31 months after the procedure, he still reported satisfactory tremor control, and the FTM was 10.

The second patient was a 68-year-old, right-handed woman, with a history of essential tremor (ET) lasting for more than 40 years (Table 2). She was admitted to our day hospital for evaluation in September 2021. In the previous six years, the tremor worsened, considerably influencing her quality of life and leading to an earlier retirement. Previous treatment included propranolol and primidone in adequate doses but without significant tremor relief. On neurological examination, severe action and postural tremors of the upper limbs were observed. The FTM score was 65. There were no other movement disorders or neurological deficits. The patient had multiple comorbidities (arterial hypertension, hypertensive cardiomyopathy, diabetes, and hypothyroidism). Brain MRI revealed multiple chronic ischemic changes of the white matter corresponding to the Fazekas 2. Neuropsychological testing revealed slight cognitive impairment and severe depression, requiring psychiatric follow-up and anti-depressive treatment.

**Table 2:** Timeline of events and interventions for case 2*

Date	Clinical information
45 years before baseline	Start of progressive bilateral hand tremor, postural tremor worsening on action. At first no treatment, afterwards small benefit with oral treatment.
6 years before baseline	Severe disability due to tremor, early retirement, unable to write and use eating utensils, forced to eat mashed food.
Baseline visit – September 2021	Severe action and postural tremor of the upper limbs, FTM 65, BFT-ADL 72. Severe depression. Brain MRI Fazekas 2.
2 months later	GK radiosurgery of the left thalamic VIM nucleus – 130 Gy 4 mm isocenter. No complications.
6 months later (4 months after GK)	Follow-up: significant improvement in right-sided tremor, FTM 28. No side effects.
10 months later (8 months after GK)	Follow-up: brain MRI (Figure 2 and 3) showing adequate left VIM lesion. No side effects.
14 months later (12 months after GK)	Follow-up: further improvement in right-sided tremor – again able to use the right hand for writing and using eating utensils. Improvement in BFT-ADL 28.

*Abbreviations: UPDRS - Unified Parkinson´s Disease Rating Scale; FTM - Fahn-Tolosa-Marin tremor rating scale; BFT-ADL - Bain and Findley tremor activities of daily life scale score; DBS – deep brain stimulation; GK – gamma knife; VIM – ventral intermediate nucleus of thalamus; MRI – magnetic resonance imaging.

The patient was classified as having refractory, disabling ET requiring further invasive treatment. Based on multiple comorbidities and patient preference, we decided to perform left-sided GK thalamotomy, applying 130 Gy radiation through a single 4 mm isocenter of radiation. The procedure was carried out in the same way as in patient 1, and no complications were recorded.

**Figure 2:**
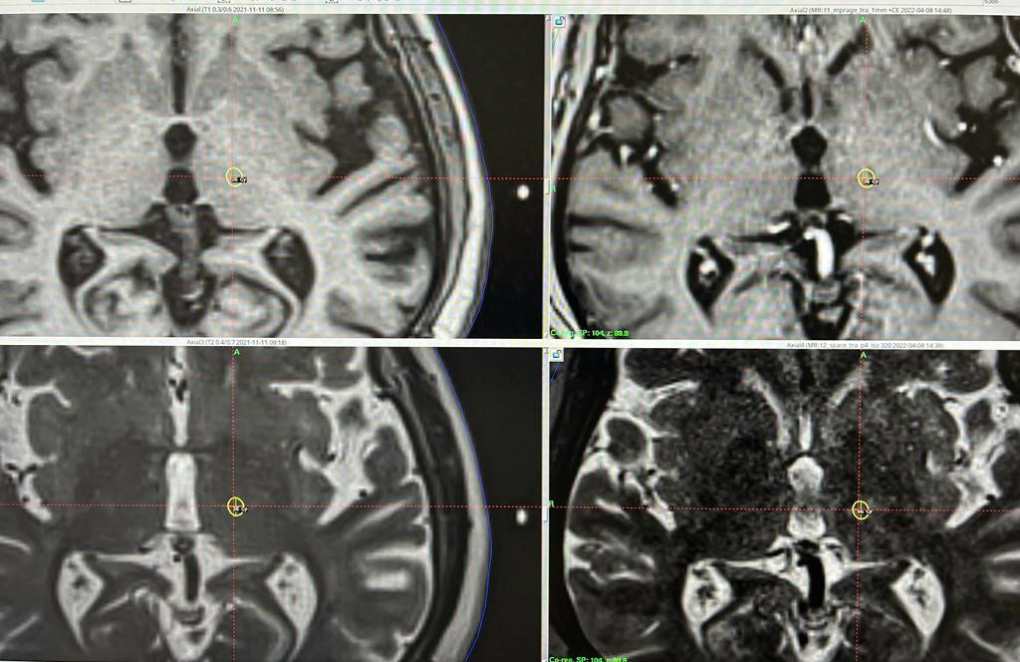
A brain magnetic resonance image (MRI) of case 2 comparing a baseline MRI (top and bottom left) to a control MRI (top and bottom right) done eight months after gamma knife thalamotomy. At the top are T1 and T1 magnetization-prepared rapid gradient echo sequences, and both sequences at the bottom are T2. An adequate irradiation lesion of the left thalamic ventral intermediate nucleus is presented.

**Figure 3:**
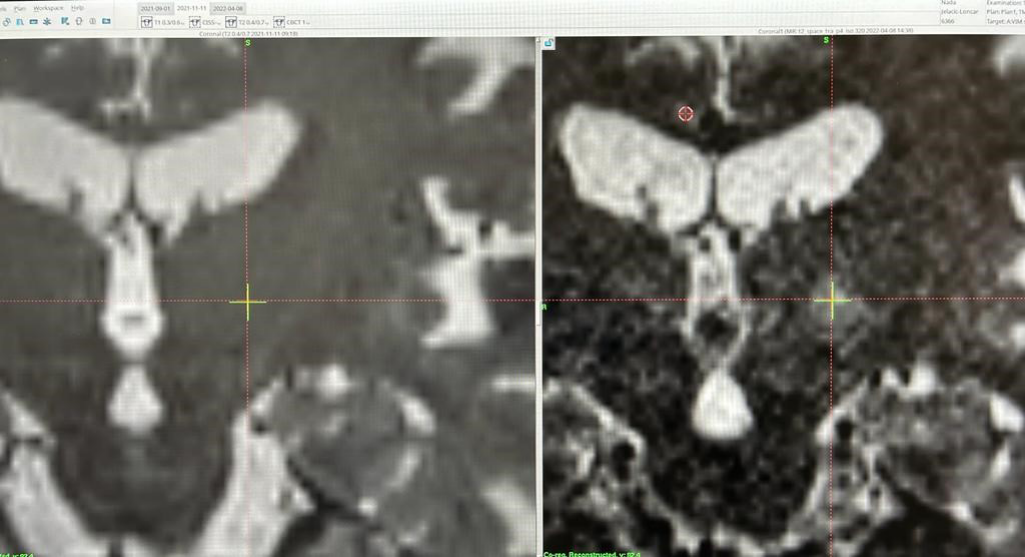
A brain magnetic resonance image (MRI) of case 2 comparing a baseline MRI coronal T2 sequence (left) to a control MRI coronal T2 sequence (right) done eight months after gamma knife thalamotomy.

On the follow-up four months later, right-sided tremor considerably improved, and the total FTM score was 28. Control brain MRI (Figure 2 and 3), performed 8 months after the procedure, showed a typical normal response lesion in the left VIM thalamic nucleus.

The improvement in right-sided tremor persisted on the last follow-up 12 months later, with no reported side effects. The patient experienced considerable improvement in her quality of life, being able to use her right arm to eat, drink, write, and perform other activities. The BFT-ADL score decreased from 72 at baseline to 28 at the end of the follow-up.

## Discussion

We present two patients with intractable parkinsonian and essential tremors who were successfully treated with GK thalamotomy. Our PD patient had a 78% improvement in tremor on the FTM rating scale sustained in the observation period of 31 months, while the patient with ET showed a 57% improvement in 12 months.

Recently published results on long-term experience with GK radiosurgery in treating essential and parkinsonian tremor in 13 patients showed improvement in FTM subscale scores: 63.6% at 12 months and 63.5% at 30 months (3). Other retrospective studies showed clinical improvement after GK thalamotomy in up to 70% to 90% of patients, mostly not showing the exact effect on tremor severity (4). Witjas et al performed a prospective study with blinded evaluation by a single neurologist in 50 patients with essential or parkinsonian tremor. They reported a global tremor improvement of 54.2% and an improvement in the quality of life by 72.2% (6). Similarly, our PD patient experienced a 68% improvement in the quality of life and our ET patient experienced 61% improvement, as assessed by the BFT-ADL score. In another prospective study, by Ohye et al, 81% of patients with essential or parkinsonian tremor showed good or excellent improvement, with no difference between different tremor groups over a period of 12 months (7). However, Lim et al reported partly negative results, with improvement seen only in the ADL of tremor patients, and no significant tremor improvement (8).

In accordance with the literature data, we performed unilateral GK thalamotomy in both patients. Most published studies used the same GK procedure (4). The initial clinical effect of the procedure was observed three months later. A delay in tremor improvement for up to one year was observed in other reports as well, and is explained by radiation-induced necrosis and subacute edema of the local tissue. The effect depends on variable radiosensitivity of the individual, with three types of reactions possible: a hypo response with no symptom relief; a normal response with the lesion confined to the desired target with symptom alleviation and without side effects; and a hyper response leading to larger necrosis and edema spreading to adjacent areas and causing more severe side effects (9). Both of our patients had a normal response observed on MR images, with clinical improvement and no side effects. Studies with a larger patient sample reported mostly transient complications of paresthesias, hemiparesis, dysarthria, and dysphagia in 1.6% to 16.7% of cases (4,7,8).

GK radiosurgery is a minimally invasive procedure with no surgical risks associated with DBS and RF ablation, producing valuable clinical benefits in treating refractory tremors. However, the treatment is available in a few medical centers worldwide with limited reported data on its long-term efficacy and tolerability. Additionally, there is no possibility to control the final clinical response because of the delayed effects of the lesion. Compared with DBS and RF thalamotomy, GK thalamotomy had slightly lower efficacy but it had a preferable side-effect profile (4,9). Recently introduced MRgFUS thalamotomy produces immediate effects and the potential for procedural target adjustments. However, it is characterized by a high incidence of paresthesias and gait disturbances, and tremor recurrence (3,10).

Here, we report the first clinical use of GK thalamotomy in Croatia to treat intractable parkinsonian and essential tremors. Favorable clinical effects and safety profiles in our patients confirm that GK thalamotomy presents a valuable treatment option in patients with contraindications for DBS.
